# Analysis of the Zn-Binding Domains of TRIM32, the E3 Ubiquitin Ligase Mutated in Limb Girdle Muscular Dystrophy 2H

**DOI:** 10.3390/cells8030254

**Published:** 2019-03-16

**Authors:** Elisa Lazzari, Medhat S. El-Halawany, Matteo De March, Floriana Valentino, Francesco Cantatore, Chiara Migliore, Silvia Onesti, Germana Meroni

**Affiliations:** 1 Department of Life Sciences, University of Trieste, 34127 Trieste, Italy; elazzari@units.it (E.L.); flovalentino@alice.it (F.V.); francesco.cantatore@studenti.units.it (F.C.); migliore.chiara81@gmail.com (C.M.); 2 Cluster in Biomedicine, Area Science Park, 34149 Trieste, Italy; m.elhalawany@gmail.com; 3 Department of Zoology, Faculty of Science, Cairo University, Cairo 12613, Egypt; 4 Structural Biology Laboratory, Elettra-Sincrotrone Trieste S.C.p.A., 34149 Trieste, Italy; matteo.demarch@elettra.eu (M.D.M.); silvia.onesti@elettra.eu (S.O.); 5 Medical Genetics, University of Siena, 53100 Siena, Italy

**Keywords:** TRIM family, E3 ubiquitin ligases, RING domain, B-box domain, Limb–Girdle Muscular Dystrophy type 2H

## Abstract

Members of the tripartite motif family of E3 ubiquitin ligases are characterized by the presence of a conserved N-terminal module composed of a RING domain followed by one or two B-box domains, a coiled-coil and a variable C-terminal region. The RING and B-box are both Zn-binding domains but, while the RING is found in a large number of proteins, the B-box is exclusive to the tripartite motif (TRIM) family members in metazoans. Whereas the RING has been extensively characterized and shown to possess intrinsic E3 ligase catalytic activity, much less is known about the role of the B-box domains. In this study, we adopted an in vitro approach using recombinant point- and deletion-mutants to characterize the contribution of the TRIM32 Zn-binding domains to the activity of this E3 ligase that is altered in a genetic form of muscular dystrophy. We found that the RING domain is crucial for E3 ligase activity and E2 specificity, whereas a complete B-box domain is involved in chain assembly rate modulation. Further, in vitro, the RING domain is necessary to modulate TRIM32 oligomerization, whereas, in cells, both the RING and B-box cooperate to specify TRIM32 subcellular localization, which if altered may impact the pathogenesis of diseases.

## 1. Introduction

Ubiquitination is a versatile form of post-translational modification that regulates a large number of processes inside the cell through the regulation of protein turnover and activity. The ubiquitination reaction is a three-step process beginning with the ATP-dependent activation of ubiquitin, a 76-amino-acid peptide, by the E1 activating enzyme. In the following step of the cascade, ubiquitin is transferred to the E2-conjugating enzyme, which then cooperates with an E3 ubiquitin ligase to transfer ubiquitin to the final target substrate [1]. Thus, the E3 ubiquitin ligase is able to provide substrate specificity to the ubiquitination cascade. In humans, the vast majority of E3 ligases possess a really interesting new gene (RING) domain, which confers catalytic activity (reviewed in [2]). Among the RING-type E3 ligases, the tripartite motif (TRIM) family represents one of the largest groups with over 70 members in humans. This class of E3 ligases is characterized by the presence of a well-conserved N-terminal motif comprising the previously mentioned RING domain, one or two B-box domains and a coiled-coil region [3]. The C-terminal domain is highly variable among different members of the family and is supposed to provide substrate specificity [4,5,6].

The RING domain of several TRIM proteins was shown to fold around two Zn^2+^ ions coordinated by conserved Cysteine and Histidine residues, while the formation of a 4-helix bundle by residues N- and C-terminal to the Zn-binding core allows for the RING dimerization necessary for ubiquitination activity [7,8]. Mechanistically, RING-type ubiquitin ligases were shown to contact the ubiquitin-loaded E2, promoting a conformational change that allows for the direct transfer of ubiquitin to the substrates without the formation of an intermediate bond between ubiquitin and the E3 ligase [9,10,11]. In this context, the dimerization of the RING domains provides the proper interaction surface to contact both the E2 and ubiquitin, ultimately allowing the discharge of ubiquitin from the loaded E2 to the substrate. In TRIM proteins, the B-box domain, C-terminal to the RING, assumes a fold similar to the latter through the coordination of Zn ions by conserved Cysteine, Histidine and Aspartate residues. The B-box domain can be present in two flavors, type 1 and 2, that have been well conserved during evolution as part of the tripartite motif, invariably located between the RING and the coiled-coil region in a unique (type 2) or tandem arranged (type 1–type 2) manner [5,12,13,14]. Whether this domain elicits or contributes to the E3 catalytic activity given its similarity with the RING domain is still controversial. Finally, in all the TRIM proteins studied, a coiled-coil region invariantly follows the B-box domain(s) and assumes a typical α-helical structure. The interaction of the coiled-coil domains of two TRIM proteins allows for the formation of anti-parallel dimers, resulting in N-terminal domains at opposed ends of the rod-like structures [15,16,17]. Therefore, the higher order self-association of dimers may be required to allow for the RING dimerization necessary for catalytic activity, as has been shown in the case of TRIM5α [18,19,20].

The TRIM32 tripartite motif is composed of a RING domain followed by a single type 2 B-box and a coiled-coil domain. The C-terminus in TRIM32 is composed of six NHL repeats (named after proteins Ncl-1, HT2A and Lin-41 containing this domain) shown to be involved in the recognition of various substrates [21]. Importantly, a cluster of mutations in the C-terminal domain or the complete deletion of the gene were shown to be associated with the neuromuscular disease Limb–Girdle Muscular Dystrophy type 2H, a recessive form of late-onset muscular dystrophy mainly affecting the proximal muscles of limbs [22,23,24]. In the disease context, TRIM32 may affect muscle physiology at many different levels, from atrophy to the regeneration of damaged fibers [21,25,26,27,28]. Interestingly, a P130S mutation in the B-box domain is associated with a non-muscular phenotype, Bardet–Biedl Syndrome (BBS), although the pathogenetic mechanism is yet to be elucidated [29]. Furthermore, like many other members of the TRIM family, TRIM32 was also shown to play a role in cancer since various oncogenes (e.g., MYCN) or tumor suppressors (e.g., p53) are among its substrates (reviewed in [21]). Given the multiple roles TRIM32 can have in both physiological and pathological conditions, understanding in detail the mechanism of action of this enzyme is important for the correct design of therapeutic strategies.

Here, we performed a biochemical analysis aimed at investigating TRIM32 cooperation with various E2-conjugating enzymes and the role of the Zn-binding domains in the regulation of its activity.

## 2. Materials and Methods

### 2.1. Constructs and Mutagenesis

The mammalian expression plasmids encoding the human TRIM32 sequence in pcDNA3X(+)-Myc-GFP and pcDNA3X(+)-HA were described previously [3]. The sequence encoding human TRIM32 was inserted in the bacterial expression plasmid, pETM11, using the restriction-free cloning method [30]. Briefly, the TRIM32 sequence was amplified using primers N1 and C5 (the full list of primers used in this study is available in Table 1), containing over-hanging tails complementary to the destination vector, and pcDNA3X(+)-Myc-GFP-TRIM32 as a template. The PCR product was subsequently purified and used as a primer pair for the second PCR reaction, which includes the destination vector as a template for a linear amplification reaction around the plasmid resulting in insertion of the sequence of interest in the vector. A primer (N2) annealing internally in the TRIM32 sequence was used in combination with the previously mentioned C5 primer to generate the mutant lacking the N-terminal 88 amino acids (TRIM32 ΔRING). Point mutations and the internal deletion of the B-box (ΔB-box, lacking residues 99–134) were generated by site-directed mutagenesis using primers listed in Table 1 and the QuikChange mutagenesis kit (Stratagene). The plasmid encoding His_6_-MBP-tagged UbE2V2 in pLou3 vector was a kind gift from Prof. Ronald T. Hay (University of Dundee).

### 2.2. Protein Expression and Purification

Plasmids (pETM11) encoding His_6_-tagged human TRIM32 (wild-type or mutated forms) were transformed into BL21(DE3) RIPL *Escherichia coli* cells. Cultures were grown at 37 °C in Terrific Broth until OD600 reached 0.6–0.8 and the expression of recombinant proteins was then induced by addition of 1 mM Isopropyl β-d-1-thiogalactopyranoside (IPTG) and 100 μM ZnCl_2_ followed by incubation at 16 °C overnight. Bacteria were lysed in lysis buffer (50 mM TRIS, pH 8; 0.5 M NaCl; 10 mM imidazole; 5 mM β-Mercaptoethanol and protease inhibitor cocktail (Roche)). Bacteria lysis was achieved by addition of lysozyme (250 μg/mL) and sarkosyl (0.3%) followed by sonication. Triton X-100 was then added to a final concentration of 0.5% before centrifugation to remove the insoluble fraction. Recombinant proteins were purified by incubation with Ni-NTA resin (Qiagen; Hilden, Germany) followed by washes with lysis buffer containing 50 mM imidazole. The resin was eluted in elution buffer (20 mM TRIS pH 8; 250 mM NaCl; 10% glycerol; 200 mM imidazole and 5 mM β-Mercaptoethanol) and the recombinant proteins were dialyzed overnight against 20 mM TRIS pH 8; 0.5 M NaCl; 10% glycerol and 5 mM β-Mercaptoethanol.

### 2.3. In Vitro Ubiquitination

Recombinant proteins used for in vitro ubiquitination assays were purchased from Boston Biochem (Cambridge, MA, USA) (UBE1 activating enzyme, UbE2D1, UbE2N), Sigma (St. Louis, MO, USA) (ubiquitin) or UBPBio (Aurora, CO, USA) (E2 screening kit). Recombinant His_6_-MBP-tagged UbE2V2 was purified as described in [10]. Ubiquitination reactions were carried out at 37 °C and contained 50 nM E1, 1 μM E2, 50 μM ubiquitin in reaction buffer (50 mM TRIS pH 7.2; 150 mM NaCl; 0.1% NP-40; 0.1 mM Tris (2-carboxyethyl) phosphine (TCEP); 3 mM ATP and 5 mM MgCl_2_). The quantity of E3 added varied in different assays and in assays where different TRIM32 mutants were employed, comparable amounts of each recombinant protein were used. The E2 screening kit was used according to the manufacturer’s instructions. Reactions were stopped by addition of Laemmli buffer containing 100 mM Dithiothreitol (DTT) final concentration.

### 2.4. Cell Culture and Transfection

The C2C12 murine myoblast cell line (kind gift from Prof. Paola D’Andrea, University of Trieste) was grown in high glucose Dulbecco’s Modified Eagle Medium (DMEM)medium without sodium pyruvate supplemented with 20% Fetal Bovine Serum (FBS), 4 mM L-glutamine, 100 U/mL penicillin and 100 μg/mL streptomycin, and maintained at 37 °C in 5% CO_2_. C2C12 cells were seeded on coverslips and transfected with HA- or Myc-GFP-tagged constructs using Lipofectamine 3000 according to the manufacturer’s instructions the day after seeding.

### 2.5. Immunofluorescence

Twenty-four to forty-eight hours post-transfection, cells were fixed in 4% Paraformaldehyde (PFA) in Phosphate Buffer Saline (PBS) for 10 min at room temperature. For immunofluorescence analysis, cells were permeabilized with 0.1% Triton X-100 in PBS and blocked with 5% Bovine Serum Albumin (BSA) in 0.1% Triton X-100 in PBS. Cells were stained with anti-HA antibody and FITC- or Cy3-conjugated secondary antibodies. Coverslips were mounted with Vectashield mounting medium with 4′,6-diamidino-2-phenylindole (DAPI) and analyzed by epifluorescent microscopy.

### 2.6. Antibodies and Immunoblot Analysis

Samples from the in vitro ubiquitination reactions were boiled and resolved by SDS-PAGE on 4–15% gradient gels (Bio-Rad). For native PAGE, proteins were prepared in Laemmli buffer lacking SDS and DTT and resolved without prior boiling on a 7.5% poly-acrylamide gel without SDS. Following transfer to PVDF membranes, target proteins were detected with anti-ubiquitin (UBPBio, clone P4D1) and anti-TRIM32 (Abcam, Cambridge, UK; ab96612), and relevant HRP-tagged secondary antibodies. Signal was detected using ECL substrate (Millipore, Burlington, MA, USA) and autoradiography films (GE Healthcare, Chicago, IL, USA). In some cases, membranes were stripped with mild stripping buffer (200 mM glycine; 0.1% SDS; 1% Tween 20; pH 2.2) prior to re-probing with a different primary antibody.

### 2.7. Quantification of In Vitro Ubiquitination Activity

Following the in vitro ubiquitination reaction as described in Section 2.3, proteins were resolved on 4–15% gradient gels (Bio-Rad; Hercules, CA, USA) followed by transfer on PVDF membranes and blotting with anti-ubiquitin antibody. For the time course in vitro ubiquitination, poly-ubiquitin chains with sizes above 3-Ub (over 25 kDa) were quantified using ImageJ and values were normalized to the quantity of E3 ubiquitin ligase used in the reaction. The resulting normalized values were plotted and the rate was calculated by dividing the normalized absorbance of poly-ubiquitin chains by the reaction time. The normalized absorbance and calculated rates are relative and have an arbitrary unit.

## 3. Results

### 3.1. Deletion or Mutation of the B-box Domain Does Not Prevent TRIM32 E3 Ligase Catalytic Activity

**Figure 1:**
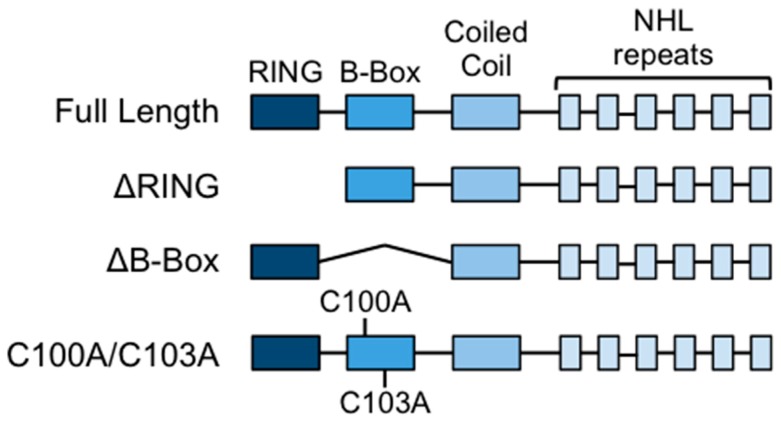
Schematic diagram showing the domain structure of TRIM32 and the deletion/point mutants used in this study.

The strictly conserved N-terminal structure of TRIM proteins suggests a potential function in catalytic activity regulation of the single domains within the tripartite module. This module may have evolved to serve as a scaffold to confer proper spacing and orientation to the catalytic RING domain and the substrate interacting domain. In the case of TRIM32, the role of the tripartite motif domains is still unclear and the available data on their contribution to E3 activity mainly rely on experiments using short protein fragments [7,31]. In this study, we focused on characterizing the role of the B-box type 2 domain of TRIM32 as a potential modulator of its activity. The B-box domain is a Zn-binding domain, assuming a fold similar to the catalytic RING domain but with a still undefined role. We generated a deletion mutant lacking the RING domain (ΔRING) to investigate whether the B-box could substitute for the RING to sustain catalytic activity (Figure 1). To further analyze the role of the B-box, we generated a mutant lacking the whole B-box domain (ΔB-box) and a mutant carrying mutations in two of the B-box cysteine residues necessary to coordinate the Zn^2+^ ions (C100A/C103A), predicting that the resulting protein would have an unfolded B-box but would maintain the spacing between the TRIM32’s RING and coiled-coil domains (Figure 1).

**Figure 2:**
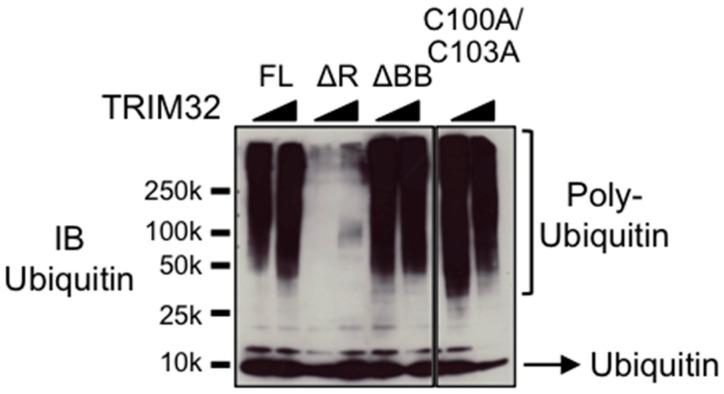
TRIM32 carrying mutations or deletion of the B-box shows activity similar to the wild-type protein. Increasing doses of recombinant full-length TRIM32 (FL), ΔRING (ΔR), ΔB-box (ΔBB) or C100A/C103A mutants were used as E3 ligase in an in vitro reaction with the UbE2D1 conjugating enzyme. Proteins were resolved by SDS-PAGE and poly-ubiquitin chains detected with anti-ubiquitin antibody.

We first assayed the activity of recombinant wild-type TRIM32 or mutants in the presence of the UbE2D1 conjugating enzyme, previously shown to cooperate with TRIM32 to build poly-ubiquitin chains [32], in the absence of specific substrates. All proteins tested but the ΔRING were able to build poly-ubiquitin chains in vitro in these experimental conditions and, strikingly, no difference was observed between the activity of the ΔB-box, the C100A/C103A and wild-type TRIM32 proteins (Figure 2). These results confirm that the catalytic activity resides in the RING domain, as already shown for other members of the TRIM family [33,34]. On the other hand, despite the structural similarity, the B-box domain cannot substitute the RING in promoting the discharge of ubiquitin from the loaded E2 as demonstrated by the lack of activity of the ΔRING mutant (Figure 2). Furthermore, these data indicate that neither mutation nor the deletion of the B-box has dramatic effects on the ability of TRIM32 to build poly-ubiquitin chains.

### 3.2. The B-box Domain Plays a Role in Modulating TRIM32 E3 Ligase Reaction Rate

**Figure 3:**
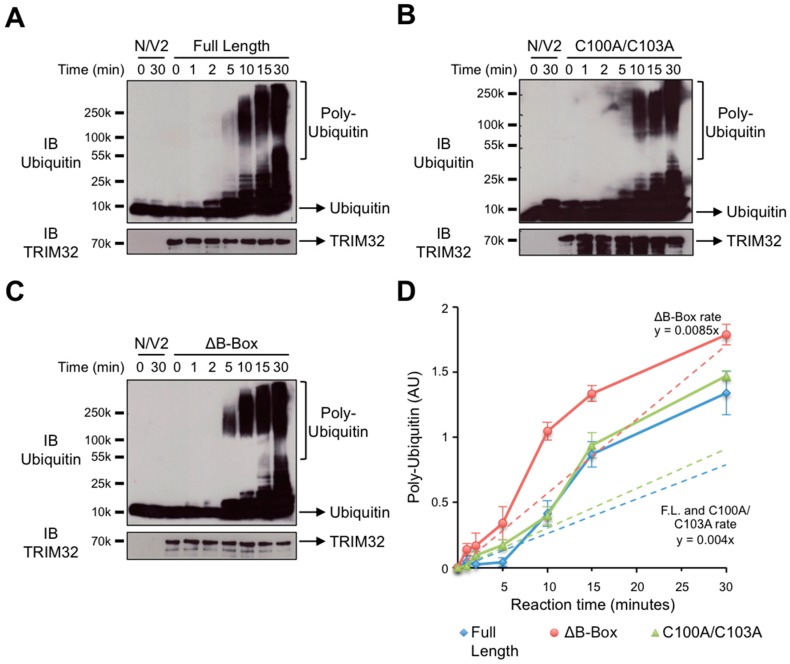
Deletion of the TRIM32 B-box domain affects the rate of poly-ubiquitin chain formation. Comparable amounts of full-length TRIM32 (**A**), C100A/C103A (**B**) or ΔB-box (**C**) mutants were used in an in vitro ubiquitination reaction with the UbE2N/V2-conjugating enzymes. Samples were taken at the indicated time points and proteins were resolved by SDS-PAGE. Poly-ubiquitin chains were detected by anti-ubiquitin immunoblot (top panels) followed by stripping and reprobing of the membrane with anti-TRIM32 antibody (bottom panels). (**D**) Poly-ubiquitin chains were quantified, normalized to the amount of E3 used in the assay and plotted (solid lines). The observed reaction rate was calculated and plotted (dashed lines). Equations showing the calculated reaction rate for each construct are shown. Average and standard error of five independent experiments are represented.

The results of the previous section show that deletion or mutation of the TRIM32 B-box domain has no effect on catalytic activity when the amount of poly-ubiquitin chains produced by the various constructs is measured at the endpoint of the reaction. It is, however, possible that the B-box, either through cooperation with the RING or simply as a spacer between the RING and the C-terminal domains, may be involved in modulating the rate at which TRIM32 builds the poly-ubiquitin chains, as it was recently shown for the coiled-coil domain of the RING-type E3 ligase, TRAF6, in the ubiquitination reaction with UbE2N/V2 [35]. We thus performed a time course experiment using UbE2N/V2, previously shown to work with TRIM32 [32], together with full-length TRIM32, ΔB-box and C100A/C103A and analyzed the progressive appearance of poly-ubiquitin chains by ubiquitin immunoblot of SDS-PAGE-resolved reaction mixtures taken at different time points (Figure 3). Confirming the previous results, no major difference was observed in the endpoint amounts of poly-ubiquitin chains produced by the different constructs with this E2 enzyme too (Figure 3, Time 30 min). Interestingly however, the analysis of earlier time points revealed that the construct lacking the B-box (ΔB-box) presents a slightly enhanced ability to build poly-ubiquitin chains when compared to the full-length protein. In particular, upon quantification of the poly-ubiquitin chains produced by the different constructs, we observed that the TRIM32 reaction rate was not affected by point mutations in the B-box, since the wild-type and C100A/C103A mutant showed comparable ability to build poly-ubiquitin chains with small amounts of high molecular weight chains that appeared after 5 min of reaction and progressively increased over time. However, when the B-box was deleted, we observed an increased reaction rate and indeed, larger amounts of poly-ubiquitin chains were produced by the ΔB-box construct at earlier time points compared to the full-length protein, either wild-type or carrying point mutations in the B-box domain (Figure 3D). Altogether, these results suggest a role for the B-box as a spacer able to modulate TRIM32 catalytic activity in vitro.

### 3.3. TRIM32 E2 Specificity Is Not Determined by the B-box

**Figure 4:**
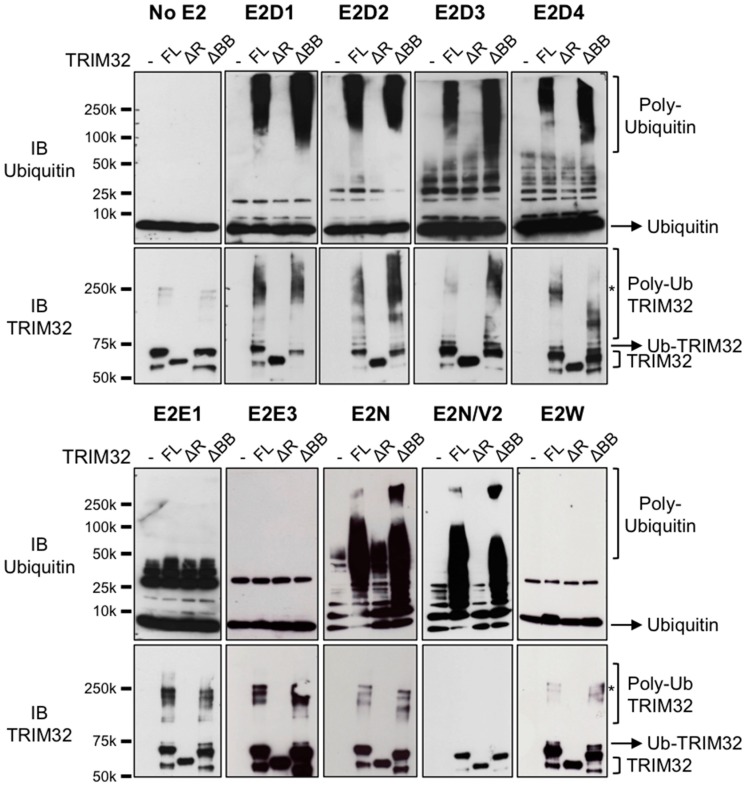
Deletion of the B-box does not prevent TRIM32 catalytic activity and does not change UbE2 specificity. Recombinant full-length TRIM32 (FL), ΔRING (ΔR) or ΔB-box (ΔBB) was used as E3 ligase in in vitro ubiquitination reactions with a panel of UbE2-conjugating enzymes as indicated. In each panel, the first lane is the reaction with no E3 (−). Control reactions without the addition of E2 enzymes are shown (No E2). Proteins were resolved by SDS-PAGE and membranes incubated with anti-ubiquitin (top panels) or anti-TRIM32 (bottom panels). Asterisks indicate SDS-resistant high molecular weight species likely representing TRIM32 oligomers (see text below).

In the RING-mediated ubiquitination process, the E2-conjugating enzyme can determine the topology of poly-ubiquitin chains on the modified substrate. However, since the latter is recognized specifically by the E3, it follows that different E2–E3 combinations will differentially affect the fate of substrates (reviewed in [36]). Previous data from our group indicate that the majority of TRIM proteins interact and work with E2s with defined specificity [32]. The results above suggest a role for the B-box in modulating TRIM32 catalytic activity, potentially by regulating the interaction between the catalytic RING domain and the ubiquitin-loaded E2. In this scenario, we next considered whether the B-box might thus modulate the E2 specificity, ultimately controlling the topology of the chains built by TRIM32. To this end, we tested the ability of recombinant full-length TRIM32 or carrying a deletion of either the RING or the B-box domain to build poly-ubiquitin chains in cooperation with 29 different E2-conjugating enzymes in vitro. Consistent with previous results and extending these data, TRIM32 was able to build poly-ubiquitin chains with E2 enzymes of the D family (UbE2D1-D4), UbE2N and UbE2N in combination with UbE2V2 (Figure 4, anti-ubiquitin immunoblot). We observed the auto-ubiquitination of TRIM32 in the presence of UbE2D-conjugating enzymes in the form of a typical ladder (Figure 4, top panels, anti-TRIM32 immunoblot). Employing UbE2N and UbE2N/V2, we observed the formation of high molecular weight species (Figure 4, bottom panels, ubiquitin immunoblots), which likely represent free ubiquitin chains [37]. Interestingly, the anti-TRIM32 immunoblot also showed that TRIM32 promotes the self-addition of a single ubiquitin moiety in cooperation with UbE2E1 and E3, and with the mono-ubiquitinating UbE2W enzyme, consistent with the assessed role for these enzymes in ubiquitination [38,39] (Figure 4, bottom panels, anti-TRIM32 immunoblot). The other assayed UbE2s did not sustain the formation of poly-ubiquitin chains, thus indicating a marked UbE2 specificity (Figure S1).

As observed in previous in vitro experiments, the deletion of the RING domain abolishes TRIM32 activity in all cases, confirming that the catalytic activity resides in this domain and that the B-box cannot mediate ubiquitination with any of the E2 tested. Of note, with all the UbE2s above, the activity of full-length TRIM32 is comparable with the ΔB-box protein when used as E3 in the reaction. No major difference was observed not only in the auto-poly-ubiquitination reactions (UbE2D1-4), but also when mono-ubiquitination (UbE2E1, E3, W) or formation of free ubiquitin chains (UbE2N, N/V2) was catalyzed (Figure 4). These data indicate that the deletion of the B-box domain does not affect TRIM32 E2 specificity or ubiquitinating ability when examined at the endpoint of the reaction. The same holds true for the UbE2s not supporting activity with the full-length TRIM32, which do not show variations when the ΔB-box mutant is employed (Figure S1). Altogether, these results indicate that the deletion of the B-box domain does not affect TRIM32 E2 specificity in vitro.

### 3.4. The RING Domain Is Involved in Higher-Order Self-Association of TRIM32

**Figure 5:**
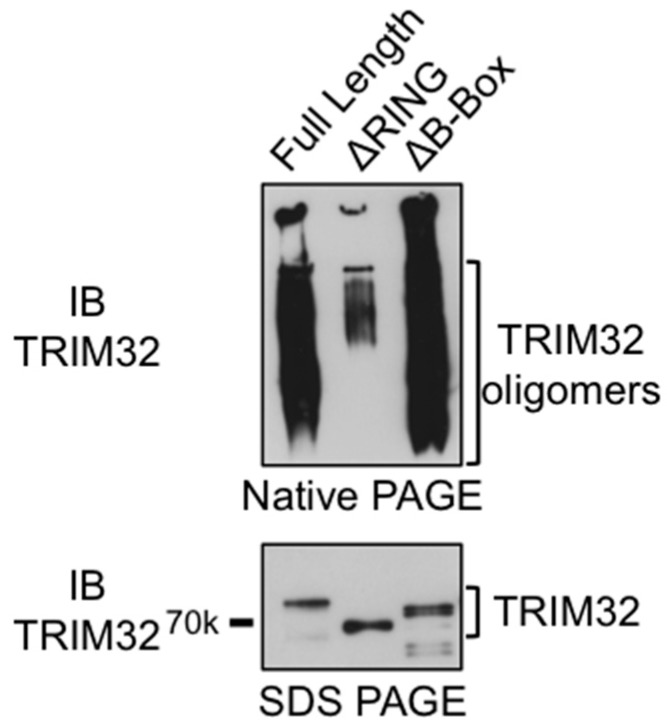
TRIM32 RING domain is necessary for oligomerization. Recombinant full-length TRIM32, ΔRING, and ΔB-box mutants were run on native PAGE (top panel) or in denaturing conditions (SDS-PAGE, bottom panel) and detected by anti-TRIM32 immunoblot.

Structural and biochemical studies have demonstrated that the coiled-coil domain in TRIM proteins is necessary for dimerization, while other domains, such as the B-box domain in the case of TRIM5α, are necessary to mediate the higher-order self-association of TRIM proteins [17,19,20]. In TRIM32, the isolated RING, as well as the RING-B-box fragment, exists as a dimer; however, the construct containing the RING, B-box and coiled-coil domains is tetrameric in solution [7]. To investigate the potential role of RING and B-box in regulating the self-association of TRIM32, we next examined the oligomerization status of TRIM32 full-length or carrying deletions of one of these domains by native PAGE electrophoresis. The results in Figure 5 show the presence of high molecular weight species formed by the oligomerization of the full-length TRIM32 protein (Figure 5). Strikingly, the formation of high molecular weight species is greatly reduced when the construct lacking the RING domain is examined, while the deletion of the B-box does not affect TRIM32 oligomerization. Indeed, SDS-resistant high molecular weight complexes were also observed in the TRIM32 immunoblots presented in Figure 4, and these were consistently not formed in the presence of the ΔRING mutant. Taken together, these observations indicate that the RING domain, rather than the B-box domain, is critical for the stabilization of higher-order oligomers in the case of TRIM32.

### 3.5. The RING and B-box Domains Contribute to Specify TRIM32 Subcellular Localization

**Figure 6:**
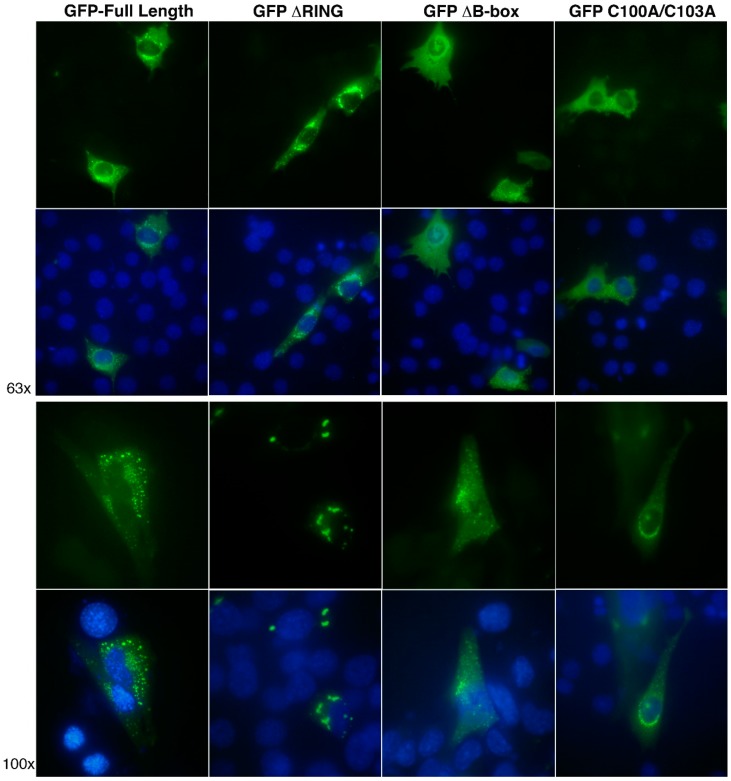
TRIM32’s RING and B-box domains contribute to determine TRIM32 subcellular localization. Representative images of C2C12 myoblasts expressing exogenous GFP-tagged full-length TRIM32 or ΔRING, ΔB-box and C100A/C103A mutants. Localization was assessed by fluorescence imaging with nuclei counterstaining (DAPI) at 63× and 100× magnification as indicated.

So far, the experiments have focused on in vitro auto-ubiquitination activity. Thus, to start characterizing the role of different TRIM32 domains belonging to the tripartite module in an in vivo context, we next analyzed the subcellular localization of TRIM32 constructs by expressing the GFP-tagged full-length, RING or B-box domain deletion mutants or the B-box point mutant in the murine myoblast cell line, C2C12 (Figure 6). Like many TRIM family members, TRIM32 was shown to form characteristic cytoplasmic bodies that are evident when expressing the full-length protein [32,40,41]. The speckles are small and distributed throughout the cytoplasm. Interestingly, the localization of the protein lacking the RING domain appears to be more perinuclear and the cytoplasmic bodies formed are larger as compared to the full-length protein. The proteins lacking the B-box or carrying mutations in the B-box Zn-coordinating residues, on the other hand, appear to have a more diffuse cytoplasmic localization with only some cells showing the typical punctate localization (Figure 6).

**Figure 7:**
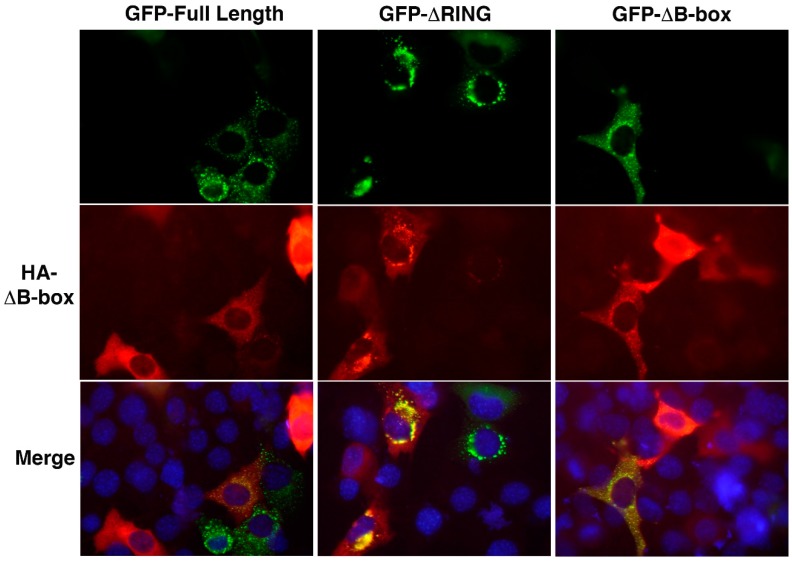
Co-localization of TRIM32 mutants. Representative images of C2C12 myoblasts co-expressing exogenous HA-ΔB-box (red signal) and GFP-tagged full-length TRIM32 or ΔRING, ΔB-box mutants (green signal). Localization was assessed by fluorescence imaging with nuclei counterstaining (DAPI) at 40× magnification.

We next examined the interplay between the different TRIM32 domains in specifying its subcellular localization by co-expressing constructs lacking the B-box with the full-length or ΔRING mutant. In keeping with previous observations, the results in Figure 7 show that constructs lacking the B-box assume a more diffuse localization within the cell, with some small cytoplasmic bodies dispersed in the cytoplasm (Figure 7, middle panels). Interestingly, the co-localization of full-length TRIM32 with the ΔB-box mutant is observed within cytoplasmic bodies; however, part of the ΔB-box protein remains diffuse in the cytoplasm even in the presence of wild-type TRIM32. Likewise, in presence of the ΔRING mutant, the protein lacking the B-box domain remains diffuse in the cytoplasm. Strikingly however, the construct lacking the RING domain is able to recruit the ΔB-box mutant in the large perinuclear structures typically formed in absence of the RING domain. Taken together, these results confirm that both the RING and B-box domains are critical in specifying TRIM32 sub-cellular localization. However, the ability of mutants lacking the N-terminal domains to localize in the same sub-cellular structures suggests that other domains, including the coiled-coil, are likely involved in mediating self-association, while the N-terminal domains may be necessary to specify sub-cellular localization by directing localization towards yet-to-be-identified specific sub-cellular structures. This in turn may represent an important mechanism to regulate interactions with specifically localized pools of substrates and relevant E2-conjugating enzymes, ultimately controlling the enzymatic activity.

## 4. Discussion

In this work, we investigated the role of the Zn-binding domains of the tripartite motif of TRIM32. Biochemical studies originally identified a role for the RING domain in TRIM proteins in promoting the direct transfer of ubiquitin from a loaded E2 to the substrate and this feature is also conserved in other RING-type E3 ligases not belonging to the TRIM family, such as Ring Finger protein 4 (RNF4) or Mouse double minute 2 (Mdm2) [42,43] and reviewed in [2]. However, a hallmark of TRIM proteins is the presence, C-terminal to the RING, of conserved B-box motifs that, in animals, are exclusive to this protein family and are predicted to assume a fold similar to the RING itself through Zn atom coordination [12,13,14]. It has been hypothesized, therefore, that the B-box domains could act as the RING domains to promote the building of poly-ubiquitin chains. Indeed, a very weak catalytic activity was observed in vitro for the RING-less TRIM member, TRIM16, and interestingly, the activity was shown to depend on the presence of the B-box domains [44]. Our in vitro analysis revealed, however, that the B-box in TRIM32 is unable to substitute for the RING to promote the building of ubiquitin chains, as already observed for other members of the family [33,45,46]. Of note, TRIM16 possesses a B-box tandem repeat formed by a B-box type 1 and a B-box type 2 domain, while TRIM32 holds a single type 2 B-box domain. Of the two domains, the type 1 B-box was shown to present a higher level of similarity to the RING domain and, furthermore, the tandem B-box repeats of another TRIM family member, MID1, were shown to form an intramolecular complex resembling a RING dimer [47]. It is therefore tempting to speculate that the intrinsic characteristics of the type 1 B-box together with the possibility to dimerize with the type 2 B-box might explain the weak activity observed for the RING-less TRIM16 and that, in other TRIM proteins possessing a single type 2 B-box, such as TRIM32, the ubiquitination relies exclusively on the presence of the RING domain.

To further characterize the potential role of the B-box, we compared the reaction rate of full-length TRIM32 with the ΔB-box and C100A/C103A constructs. One previous report indicated the possibility that the C-terminal NHL domain in TRIM32 is involved in binding of UbE2N [48]. While, as mentioned previously, the interaction of the loaded E2 with the RING is necessary for the ubiquitination reaction, binding of the E2 to domains other than the RING may be a mechanism to increase the local concentration of ubiquitin-loaded E2s and may thus affect the processivity of the reaction. Indeed, such a mechanism was shown to be employed by the E3 ligase TRAF6 that, upon oligomerization, binds the ubiquitin-loaded E2s with its coiled-coil domain, thus increasing their availability for the RING domain to achieve the rapid building of long poly-ubiquitin chains [35]. In this scenario, we hypothesized that the ΔB-box construct, having a reduced distance between the NHL and the RING, may present an increased reaction rate compared to the C100A/C103A mutant, which retains the full B-box, albeit mutated. Indeed, a comparison of the time-dependent production of poly-ubiquitin chains generated by the full-length, ΔB-box and C100A/C103A constructs revealed that the protein lacking the B-box is slightly more efficient in building poly-ubiquitin chains when compared to the full-length and the B-box point mutant, in particular in the first stages of the reaction. On the other hand, point mutation of the B-box resulted in an activity and reaction rate comparable to the wild-type protein. Therefore, our results indicate that the B-box may also act as a spacer to regulate TRIM32 processivity. However, the exact mechanism and the potential involvement of domains other than the RING in interaction with the ubiquitin-loaded E2 need further study.

We also assessed the potential involvement of the B-box in determining E2 specificity. In our analysis, the protein carrying a deletion of the B-box showed the same E2 specificity of the full-length one, indicating that the B-box is likely not involved in interaction with the E2. Indeed, previous studies have shown that the RING domain of TRIM32 is strictly necessary for interaction with UbE2N, while a TRIM32 mutant lacking the B-box domain maintains the interaction in a Yeast Two-Hybrid setting, further indicating that the B-box is not necessary for association with the E2 [32]. The inability of TRIM32 B-box to interact with the E2 also potentially explains its lack of activity, since interaction with the ubiquitin-loaded E2 was shown to be at the basis of the conformational change necessary to promote the discharge of ubiquitin from the E2 active site (reviewed in [49]). However, we cannot exclude that the B-box may bind the nascent ubiquitin chain. In this scenario, it would be intriguing to investigate whether the B-box may play a role, together with the E2, in determining the topology of the ubiquitin chain. Furthermore, experiments presented in this study did not investigate the possibility that the B-box may be involved in substrate recognition, as is the case for the B-boxes in MID1 [50], thus the role of TRIM32 B-box in the context of substrates modifications awaits further studies.

The TRIM5α B-box domain was shown to be necessary to promote the formation of the lattice required for HIV capsid recognition and for the dimerization of the RING domains necessary for activity [15,19]. Likewise, Small-angle X-Ray Scattering (SAXS) analysis revealed that TRIM32 forms tetramers in solution deriving from the juxtaposition of two dimers formed through coiled-coil interactions [7]. To our surprise, while the B-box is dispensable for the formation of TRIM32 oligomers in vitro, these are greatly reduced in absence of the RING domain, which must thus play a critical role in the stabilization of oligomers. Indeed, the RING domain of TRIM5α was also shown to contribute significantly to self-association [51].

Within a cellular context, however, analysis of the subcellular localization revealed that both the RING and B-box are involved in determining TRIM32 localization, as already observed for other members of the TRIM family [3]. The typical cytoplasmic bodies formed by the oligomerization of wild-type TRIM32 appear in fact to be altered when the RING or B-box domains are absent or mutated, with the lack of the RING leading to the formation of larger aggregates less dispersed in the cytoplasm while the lack of B-box, on the other hand, results in the reduced formation of aggregates/cytoplasmic bodies, indicating that both the RING and B-box domains play a role in determining TRIM32 sub-cellular localization in vivo. Of note, both a complete deletion of the B-box and a 2-residue-substitution likely leading to an improperly folded domain, despite a different behavior shown in in vitro ubiquitination, have the same effect on TRIM32 localization. This might suggest different roles of this conserved domain in biochemical activity and in cellular distribution. Interestingly, a domain similar to the B-box was identified in the deubiquitinase enzyme CYLD, where it was also shown to be necessary to determine its cytoplasmic localization probably through interactions with cytosolic proteins [52]. Likewise, upon co-expression, we were able to observe the co-localization of mutants lacking either the RING or B-box domain, indicating that these deletion mutants retain the ability to self-associate. However, altered localization of the ΔRING and ΔB-box mutants suggests the possibility that these domains may specify TRIM32 subcellular localization by mediating interactions with specific subcellular structures. Furthermore, since cytoplasmic body formation is a dynamic process which regulates the pool of soluble and active TRIM32 present in cells and thus the interaction with partner proteins, the RING and B-box domains therefore assume a critical role in the regulation of this process, ultimately controlling TRIM32 activity [53]. In future studies, it will be interesting to assess the composition of these cytoplasmic compartments in terms of ubiquitination machinery components and specific disease-related substrates.

## Figures and Tables

**Table 1:** List of primers used in this study. For mutagenizing primers, bases bordering the deletion (ΔB-box) or corresponding to the mutations (C100A/C103A) are in red.

TRIM32	Forward (N1)	5′-gagaatctttattttcagggcgccatggctgcagcagcagcttctcac-3′
	Reverse (C5)	5′-caagcttgtcgacggagctcgaattcctatggggtggaatatcttctcagatggta-3′
TRIM32 ΔRING (Δ1–88)	Forward (N2)	5′-gagaatctttattttcagggcgccgctgggctcagcgaggctgtggggc-3′
	Reverse (C5)	5′-caagcttgtcgacggagctcgaattcctatggggtggaatatcttctcagatggta-3′
TRIM32 ΔB-box (Δ99–134)	Forward	5’-agctgcttctttgacagggagtggcagcagccccacagcctcgctgag-3’
	Reverse	5’-ctcagcgaggctgtggggctgctgccactccctgtcaaagaagcagct-3’
TRIM32 C100A/C103A	Forward	5’-cagacgccgcccagcggaccgagccatgagcagcccc-3’
	Reverse	5’-ggggctgctcatggctcggtccgctgggcggcgtctg-3’

## Supplementary Materials

The following are available online at https://www.mdpi.com/2073-4409/8/3/254/s1. Figure S1: E2-conjugating enzymes showing no activity in combination of full-length TRIM32, ΔRING or ΔB-box.
