# Supplementary material for: Analysis of the Zn-Binding Domains of TRIM32, the E3 Ubiquitin Ligase Mutated in Limb Girdle Muscular Dystrophy 2H
Source: Cells. 2019 Mar 16;8(3):254. doi: 10.3390/cells8030254 (PMC6468550; doi:10.3390/cells8030254)
Supplement: Supplementary file 1 [file cells-08-00254-s001.pdf]

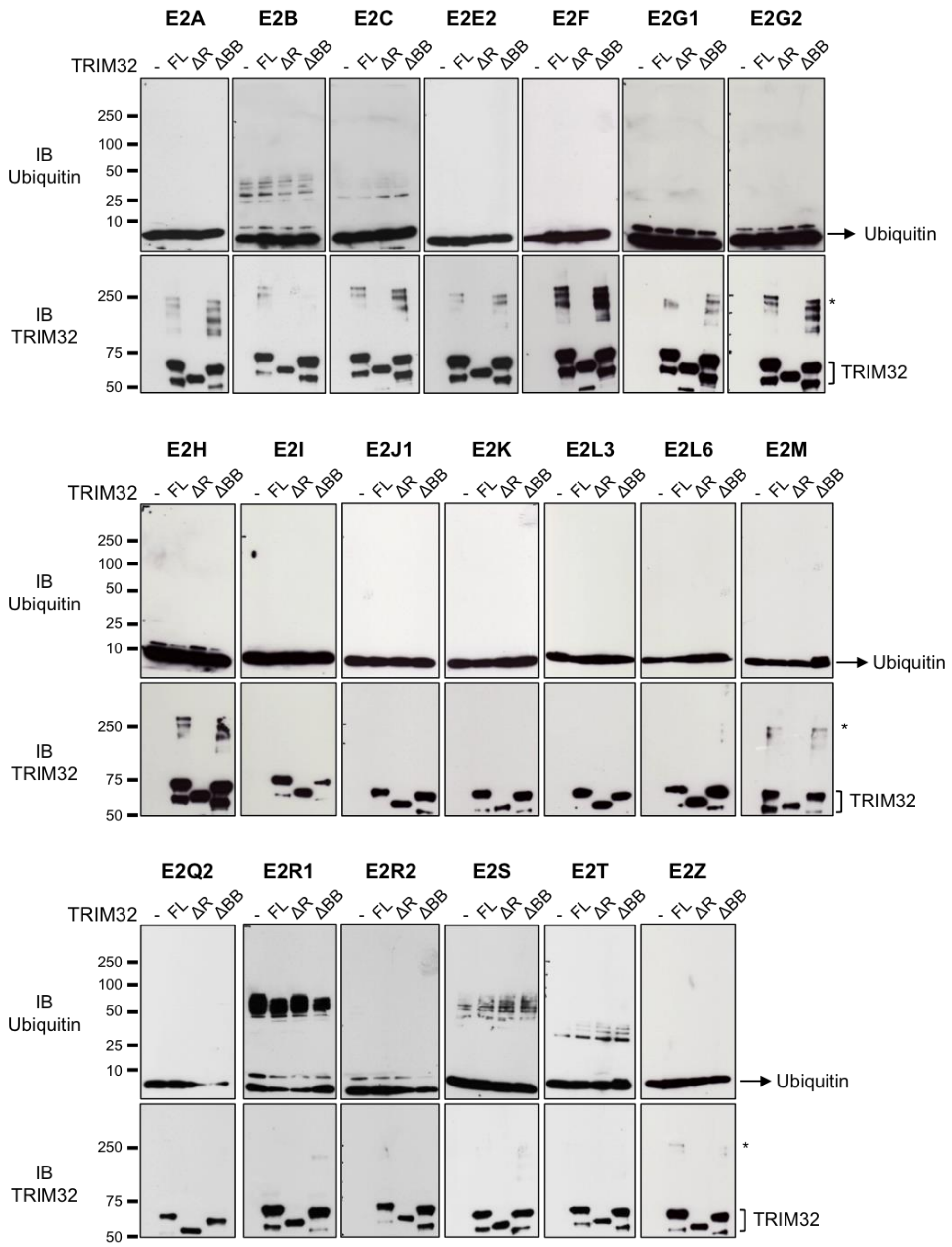

Figure S1. E2 conjugating enzymes showing no activity in combination of TRIM32 full-length, ΔRING or ΔB-Box. Recombinant TRIM32 full-length (FL), ΔRING (ΔR) or ΔB-Box (ΔBB) was used as E3 ligase in *in vitro* ubiquitination reactions with a panel of E2 conjugating enzymes. In each panel the first lane is the reaction with no E3 (-). Control reactions without the addition of E2 enzymes are shown (No E2). Proteins were resolved by SDS-PAGE and membranes incubated with anti-Ubiquitin (top panels) or anti-TRIM32 (bottom panels). Asterisks indicate SDS-resistant high molecular weight species likely representing TRIM32 oligomers (see text).
